# Resmetirom in the Management of Metabolic Dysfunction-Associated Steatohepatitis (MASH): A Comprehensive Review of Current Evidence and Therapeutic Potential

**DOI:** 10.7759/cureus.74772

**Published:** 2024-11-29

**Authors:** Raj H Patel, Charmy Parikh, Henil Upadhyay, Sneh Sonaiya, Parameswaran Ramnath, Shubham Singh, Umang Patel, Truptesh Kothari

**Affiliations:** 1 Internal Medicine, St. Mary Medical Center, Langhorne, USA; 2 Internal Medicine, Mercy Catholic Medical Center, Philadelphia, USA; 3 Respiratory Medicine, Newcastle Upon Tyne Hospitals NHS Foundation Trust, Newcastle upon Tyne, GBR; 4 Internal Medicine, Kirk Kerkorian School of Medicine, University of Nevada, Las Vegas, USA; 5 Medical School, Jonelta Foundation School of Medicine, Manila, PHL; 6 Medicine, Barking, Havering and Redbridge University Hospitals NHS Trust, London, GBR; 7 Gastroenterology, University of Rochester, Rochester, USA

**Keywords:** cirrhosis, fatty liver disease, mash, masld, metabolic dysfunction-associated steatohepatitis (mash), metabolic dysfunction-associated steatotic liver disease (masld), nash, nonalcoholic fatty liver disease (nafld), non alcoholic steatohepatitis (nash), resmetirom

## Abstract

Resmetirom is a thyroid hormone receptor agonist that has been recently approved by the FDA for the management of metabolic dysfunction-associated steatohepatitis (MASH). MASH is a severe form of metabolic dysfunction-associated fatty liver disease (MASLD), which is marked by hepatic inflammation and potential progression to cirrhosis and liver cancer. This review analyzes and demonstrates the efficacy of resmetirom in reducing intra-hepatic lipids, improving liver histology, and improving metabolic parameters. Key outcomes of this study indicate that resmetirom leads to non-alcoholic steatohepatitis (NASH) resolution and fibrosis improvement in a substantial percentage of patients. Although this drug has common gastrointestinal side effects, it maintains a favorable safety profile. With the increase in demand for treatments of MASH, the ongoing phase 3 trials will provide critical insights into the long-term efficacy and safety profile of resmetirom and further solidify its potential as a groundbreaking therapeutic option for the management of MASH.

## Introduction and background

Non-alcoholic steatohepatitis (NASH) or metabolic dysfunction-associated steatohepatitis (MASH), which is currently classified as metabolic dysfunction-associated steatohepatitis, represents an advanced-stage of non-alcoholic fatty liver disease (NAFLD), also known as metabolic dysfunction-associated fatty liver disease (MAFLD). MAFLD is characterized by hepatic inflammation and hepatocyte damage resulting from lipid accumulation, which can progress to fibrosis and cirrhosis [1,2]. The literature underscores the progressive nature of MASH, which can lead to liver fibrosis, cirrhosis, and potentially hepatocellular carcinoma, affecting a significant proportion of patients [3,4]. Statistical estimates indicate that approximately 24% of the adult population in the United States is affected by MASLD, with rates of progression to NASH ranging from 1.5% to 6.5% [5]. MASH poses a substantial global burden and is recognized as the predominant chronic liver disease in Western populations. The market for MASH treatments is projected to grow substantially, reaching an estimated value of $27.2 billion by 2029, driven by a marked increase in the patient population [6]. Non-pharmacological interventions for MASH management include lifestyle modifications such as dietary adjustments, including low-fat and calorie-restricted diets, increased physical activity, and weight loss promotion [7].

Pharmacological interventions for MASH comprise a variety of medical treatments targeting different aspects of the disease. Insulin sensitizers like metformin and pioglitazone reduce inflammation and hepatic fat accumulation [1]. Vitamin E acts as an antioxidant, safeguarding liver cells from damage [8]. GLP-1 receptor agonists, including liraglutide and semaglutide, enhance metabolic and hepatic health [9]. Farnesoid X receptor (FXR) agonists, particularly obeticholic acid, effectively reduce hepatic steatosis and fibrosis, although their long-term safety profile is currently under investigation [10]. Despite the promise of these treatments, further research is needed to fully understand their safety and efficacy. The focus of this article is to highlight resmetirom, a recently FDA-approved medication. Resmetirom is a thyroid hormone receptor (THR) agonist that has demonstrated significant potential in reducing intra-hepatic lipids by enhancing mitochondrial β-oxidation and improving hepatocyte mitochondrial function in MASH patients [11]

In this comprehensive review, we gathered data from relevant clinical trials and observational studies to evaluate the safety and efficacy of resmetirom in treating MASH and liver fibrosis.

## Review

Methodology

**Figure 1:**
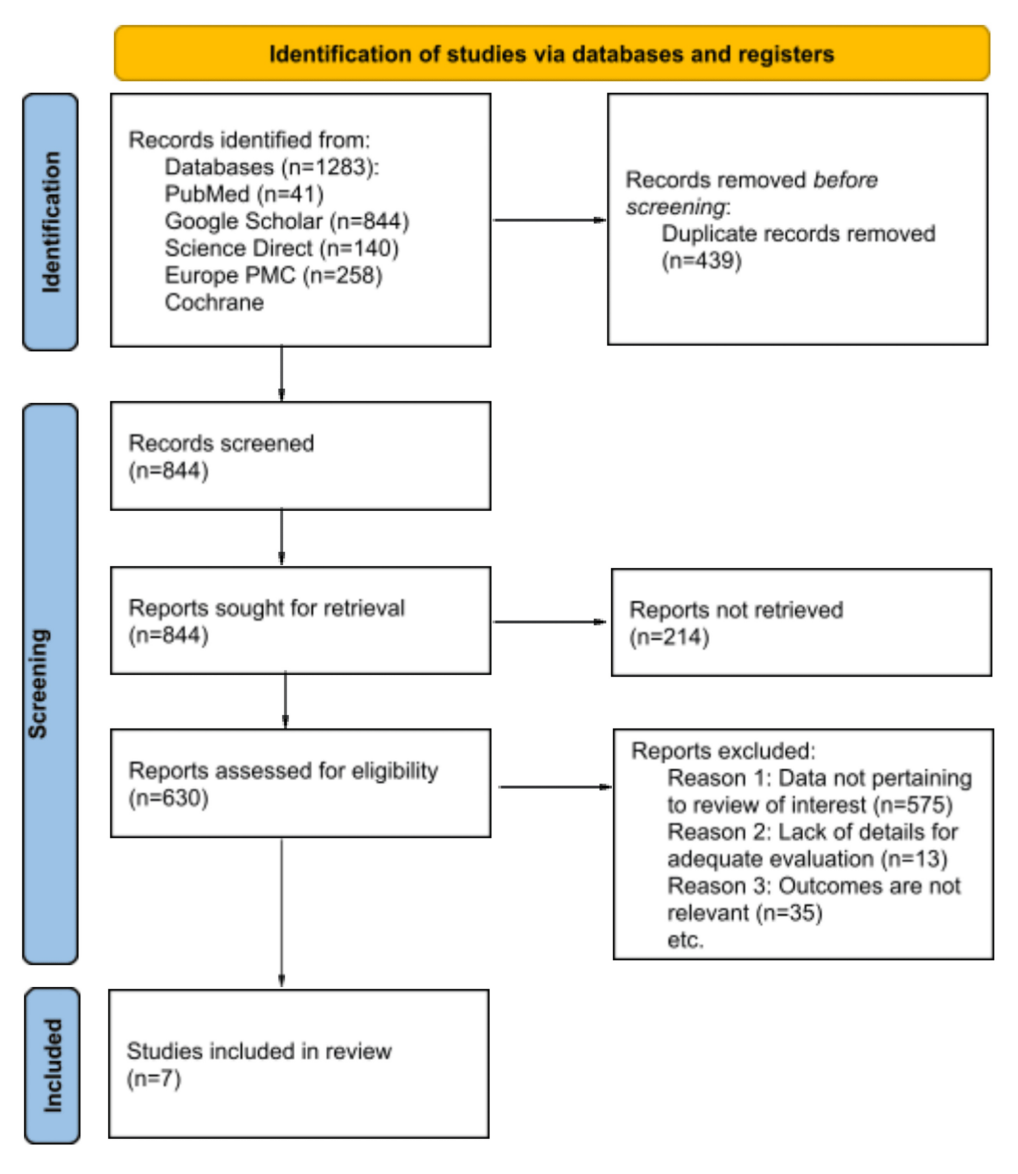
PRISMA flow diagram PRISMA: Preferred Reporting Items for Systematic Reviews and Meta-Analyses

This review focuses on the clinical outcomes reported in randomized controlled trials (RCTs) investigating the efficacy of resmetiorm in treating NASH/MASH. A systematic literature search was conducted across multiple databases, including PubMed (41 articles), Google Scholar (844 articles), Science Direct (140 articles), Europe PMC (258 articles), and Cochrane. The relevant keywords included “NASH” [OR] “MASH” [OR] “NAFLD” [OR] “MASLD” [AND] “Resmetirom”. This search initially yielded 1,283 articles. Following the removal of duplicates, a preliminary screening was conducted based on titles and abstracts. Subsequently, a detailed full-text review was undertaken to ascertain eligibility based on predefined criteria. We excluded studies involving non-human subjects, non-English publications, and those with participants below 18 years of age. Conversely, studies were included if they focused on patients diagnosed with NASH/NAFLD/MASH/MASLD through clinical or objective means and those who underwent resmetirom treatment. Adhering to the Preferred Reporting Items for Systematic Reviews and Meta-Analyses (PRISMA) guidelines [12], we ultimately selected seven RCTs for inclusion highlighted in Figure 1 [13-19].

The principal objective of this study is to evaluate the clinical outcomes reported in MASH cases after resmetirom treatment. Our emphasis was on understanding potential variations in outcome reporting across trials, rather than on the quality of the trials. Consequently, a formal quality assessment was deemed unnecessary, and our focus remained on the distinct outcomes delineated in each study. The data extracted from the selected articles encompassed various aspects, including study design, trial phase, methodology, geographical location, participant demographics, treatment duration, clinical outcomes, and adverse event profiles. Notably, we did not make a distinction between primary and secondary outcomes.

Results

Our comprehensive review included seven RCTs assessing the efficacy and safety of resmetirom in patients with MASH/NASH. Studies included Phase 2 (Table 1) and Phase 3 trials (Table 2), with participants across a range of demographics and disease severity. The clinical outcomes focused mainly on hepatic fat reduction, histological improvement, and safety profiles.

**Table 1:** Phase 2 Clinical Trials Investigating the Efficacy and Safety of Resmetirom in Patients with Non-Alcoholic Steatohepatitis (NASH) PDFF: proton density fat fraction; HRQOL: health-related quality of life

Trial/Authors, year	Design	Patients Enrolled	Treatment arms	Primary endpoints	Key Findings	Reported adverse events	Time period
Phase 2 (NCT02912260)/Harrison et al., 2019 [13]	Phase-2, double-blind, randomized controlled trial	125 patients with biopsy-confirmed NASH	Resmetirom 80 mg once daily vs Placebo	Change in hepatic fat fraction from baseline at 12 weeks	The relative reduction in hepatic fat fraction: Resmetirom vs Placebo (-32.9% vs -10.4% at 12 weeks); Improvement in histopathological features of NASH	Gastrointestinal symptoms (diarrhea, nausea), headaches, urinary tract infections, dizziness, fatigue	12 weeks
Open-Label Extension (OLE) of NCT02912260/Harrison et al., 2021 [14]	Extension study	Patients completing 36 weeks of the main trial or with predicted incomplete response	Resmetirom 80 mg initially, adjusted to 80 mg or 100 mg	Safety profile; Response to resmetirom	Well-tolerated; No increase in gastrointestinal adverse effects	Not Reported	36 weeks
Younossi et al., 2022 [15]	Phase-2, double-blind, randomized controlled trial	125 NASH patients	Resmetirom 80 mg vs matched placebo	Relative change in MRI-PDFF hepatic fat at week 12	Remetirom improved hepatic fat fraction with improvement in HRQOL scores	Not Reported	36 weeks

**Table 2:** Phase 3 Clinical Trials Evaluating the Efficacy and Safety of Resmetirom in Patients with NASH and NAFLD NASH: non-alcoholic steatohepatitis; NAFLD: non-alcoholic fatty liver disease; TEAE: treatment-emergent adverse event

Trial/Authors, year	Design	Patients Enrolled	Treatment Arms	Primary Endpoints	Key Findings	Adverse Events	Time Period
MAESTRO-NASH (NCT03900429)/Harrison et al., 2024 [16]	Phase 3, double-blind, placebo-controlled	966 patients with fibrosis staging F1B, F2, F3	Resmetirom 80 mg or 100 mg once daily vs Placebo	Resolution of NASH, reduction in liver fibrosis, prevention of disease progression	Resmetirom is superior to Placebo in NASH resolution and fibrosis improvement	Increased gastrointestinal side effects (nausea, diarrhea) in Resmetirom groups	52 weeks
MAESTRO-NAFLD-1 (NCT04197479)/Harrison et al., 2023 [17]	Randomized, double-blind, placebo-controlled	1413 patients	Resmetirom 80 mg or 100 mg vs Placebo	Incidence of Treatment-Emergent Adverse Events (TEAEs)	Resmetirom is well-tolerated; Increased gastrointestinal side effects (nausea, diarrhea) compared to Placebo		52 weeks
MAESTRO-NAFLD-OLE (NCT04951219) [18]	Open-label, Phase 3 extension	Patients completing MAESTRO-NAFLD-1 or screen failing MAESTRO-NASH/MAESTRO-NASH-OUTCOMES	Resmetirom 80 mg or 100 mg once daily	Safety endpoints	Currently recruiting; Focus on safety (TEAEs, serious adverse effects)		52 weeks
MAESTRO-NASH-OUTCOMES (NCT05500222) [19]	Multi-national, double-blind, placebo-controlled	Ongoing, estimated completion in 2027	Resmetirom 80 mg once daily	Time to experience Composite Clinical Outcome events	Recruiting; Aimed at evaluating efficacy in NASH cirrhosis patients		Estimated completion in 2027

Discussion

The clinical trials investigating resmetirom in MASH patients provide valuable mechanistic insights into its mode of action and therapeutic potential. Resmetirom is characterized by its ß-selectivity and its function as an agonist for THRs, administered orally once daily. Its primary mode of action operates through the liver, where it acts as an active oral agent targeting THRs [20,21]. Resmetirom has remarkable 28-fold selectivity for THR subtype β (THR-β) over subtype α (THR-α), and it primarily binds to liver proteins (>99%) and exhibits limited transmission to other tissues [21]. Its absorption is notably specific to the liver, facilitated by the heightened presence of THR-β receptors within hepatocytes. These receptors play a crucial role in maintaining hepatic metabolic pathways, which are often impaired in conditions such as NASH/MASH and NAFLD/MASLD. The heightened selectivity for THR-β in MASH offers a potential advantage by mitigating the systemic effects associated with excessive thyroid hormone activity in organs like the heart and bones, where THR-α predominates. Through this selective action, Resmetirom still enables the liver to leverage the metabolic benefits of thyroid hormone, potentially offering therapeutic benefits in hepatic disorders while minimizing adverse effects elsewhere in the body [22].

Phase 2 Clinical Trials Evaluating Resmetirom in NASH Patients

Harrison et al. conducted a phase-2, double-blind, randomized controlled trial (NCT02912260) at 25 centers in the United States wherein patients with biopsy-confirmed MASH and a hepatic fat fraction of at least 10% at baseline were enrolled in the study. This diagnosis was based on a positive metabolic syndrome and elastography results suggestive of liver fibrosis and steatosis. The exclusion criteria consisted of patients having a history of significant alcohol consumption or use of drugs associated with MASLD, hypothyroidism, uncontrolled diabetes mellitus type-2, or receiving glucagon-like peptide analog. Patients with other causes of liver cirrhosis, hepatic decompensation, or chronic liver disease were also excluded [13]. 

Harrison et al.'s study was aimed at dose exploration based on the adaptive exposure-based dosing scheme as phase-1 studies have demonstrated that daily resmetirom 50-100 mg had led to a statistically significant lowering of lipid. Patients were randomized to receive either 80 mg once daily resmetirom or matching placebo in a 2:1 ratio. The primary endpoint in this study was the change in hepatic fat fraction from baseline at 12 weeks in both groups. This was calculated using MRI-proton density fat fraction (PDFF) [13].

Out of 384 screened individuals in their study, 125 were randomized to receive either resmetirom (n=84) or placebo (n=41). Significant relative and absolute hepatic fat fraction reduction was seen in patients receiving the active drug as compared to placebo. Patients treated with resmetirom showed a relative reduction of hepatic fat as compared to the placebo at the end of week 12 (-32·9% resmetirom vs -10·4% placebo; least squares mean difference -22·5%, 95% CI -32·9 to -12·2; p<0·0001) and at week 36 (-37·3% resmetirom (n=74) vs -8·5 placebo (n=34); -28·8%, -42·0 to -15·7; p<0·0001). These results were also seen at week 36. More patients in the resmetirom group had a relative fat reduction of at least 30%. In addition, more patients in the high-exposure group patients met the goal of at least 30% fat reduction at 12 weeks (n=33, 75%) and 36 weeks (n=32, 74%). In terms of histopathological changes at week 36, the proportion of subjects who had at least a two-point reduction in NAS (non-alcoholic fatty liver disease activity score) and at least a one-point reduction in ballooning or inflammation was higher in the active treatment group (n=28, 46%) compared to the placebo (n=5, 19%) [13].

The most common adverse effects seen in both groups were GI symptoms such as diarrhea and nausea. GI side effects were self-limited and did not lead to any study withdrawals. Other adverse effects included headaches, urinary tract infections, dizziness, and fatigue. However, these non-GI side effects were seen in less than 15% of patients in the resmetirom group [13]. The incidence of GI adverse events drastically reduced after week 12 as noted in Table 3. This could be due to patients developing tolerance to resmetirom over time. In summary, resmetirom 80 mg was associated with a statistically significant reduction in hepatic fat and a significant improvement in MASH on liver biopsy as compared with the placebo group. Higher doses and exposure to resmetirom were associated with a reduction in NAS [1].

**Table 3:** Adverse events recorded in a phase-2 clinical trial by Harrison et al. Reference: [13]

Adverse event	Time	Resmetirom (n=84)	Placebo (n=41)
Nausea	Baseline to week 12	12 (14%)	2 (5%)
	Week 12-36	5 (6%)	1 (2%)
Diarrhea	Baseline to week 12	28 (33%)	3 (7%)
	Week 12-36	3 (4%)	1 (2%)

In 2021, Harrison et al. conducted an open-label extension (OLE) study of NCT02912260 in patients who completed 36 weeks of the main trial and those who were predicted to have an incomplete response to either placebo or resmetirom treatment in the main study [14]. This OLE study assessed the response to 80 mg and 200 mg once daily dosing of resmetirom with an emphasis on the safety profile of the drug. Patients were initially prescribed 80 mg resmetirom at the beginning of the study and based on post-dose pharmacokinetic assessment at week 2, the dose was either kept the same, up-titrated, or down-titrated by 20 mg at week 4. This study [14] was aimed at exploring the effects of resmetirom in patients who had an inadequate response to resmetirom/placebo in the primary study [13]. At week 36, MRI-PDFF reduction in both groups (80 and 100 mg) was -11.1% and -52.3% respectively. In addition, there was a decrease in markers of fibrosis, LDL cholesterol, apolipoprotein B, and triglycerides from baseline [14]. Resmetirom was well-tolerated in this study at both 80 mg and 100 mg doses. There was no increase in gastrointestinal adverse effects in the OLE study as compared to the placebo group of the main (NCT02912260) study.

Younossi et al. conducted a phase 2 double-blind clinical trial wherein 125 patients were randomized to either resmetirom 80 mg (n=84) or a matched placebo (n=41) [15]. A total of 47 patients in the resmetirom group and seven patients in the placebo group showed at least a 30% reduction in PDFF at the end of week 12. Treatment with resmetirom was associated with improvement in health-related quality of life (HRQOL) scores and this improvement continued with longer treatment. Subjects who achieved the primary efficacy endpoint showed an improvement in HRQOL scores, irrespective of the treatment arm. This was the only phase-2 clinical trial assessing the HRQOL in patients with MASH treated with resmetirom. However, one of the limitations of this study was the limited sample size and about half the patients being treated with a suboptimal dose (60 mg) of resmetrirom [15]. 

Phase 3 MAESTRO Clinical Trials Programme

Currently, four phase-3 clinical trials are evaluating the efficacy and safety of resmetirom in NASH patients. These trials have been set up with a specific goal including pivotal serial liver biopsy/outcomes (MAESTRO-NASH trial), safety and biomarker changes (MAESTRO-NAFLD-1, MAESTRO-NAFLD-OLE), and second pivotal outcomes trials in adults with well-compensated NASH cirrhosis (MAESTRO-NASH-OUTCOMES). The MAESTRO-NASH, MAESTRO-NAFLD-1, and MAESTRO-NAFLD-OLE focus on patients with non-cirrhotic NASH [16-19,23]. 

The MAESTRO-NASH is a 54-month long Phase 3 double-blind placebo-controlled study that will evaluate the efficacy of 80 or 100 mg of resmetirom in resolving NASH and/or reduction in liver fibrosis and/or preventing progression to cirrhosis/advanced liver disease. The primary endpoints of this trial are the resolution of MASH and a two-point reduction in NAS with no worsening of fibrosis; or improvement in fibrosis by more than 1 stage with no worsening of MASH. Patients will be randomized to three arms in a 1:1:1 ratio (resmetirom 80 mg and 100 mg vs placebo administered once daily) [23].

MAESTRO-NASH clinical trial (NCT03900429): A total of 966 patients who had fibrosis staging of F1B, F2, and F3 at baseline were randomized in this trial: resmetirom 80 mg (n=322); resmetirom 100 mg (n=323) and placebo (n=321). Patients who had fibrosis F1A or F1C staging at baseline (n=84) were similarly randomized to receive 80 mg vs 100 mg vs placebo once daily (n=30, 26, and 28 respectively) [23]. At the end of the trial, 25.9% of patients in the 80 mg resmetirom arm and 29.9% of patients in the 100 mg resmetirom arm achieved NASH resolution with no worsening of fibrosis. However, only 8.7% of patients in the placebo group achieved MASH resolution at the end of 52 weeks. Of patients in 80 mg and 100 mg resmetirom groups, 24.2% and 29.3% respectively achieved fibrosis improvement (by at least one stage). Only 14.2% of patients in the placebo group achieved this target [16].

GI side effects such as diarrhea and nausea were more common in the resmetirom group as compared to the placebo. This is consistent with the results from phase 2 clinical trials. The median duration of these diarrhoea episodes was 15-20 days irrespective of the dose of resmetirom received. However, more patients (6.8%) in the 100 mg resmetirom group discontinued treatment due to adverse effects as compared to the other groups (1.8% and 2.2% in the 80 mg resmetirom and placebo group, respectively) [23]. The MAESTRO-NASH trial showed that both 80 and 100 mg resmetirom was superior to placebo in terms of MASH resolution and improvement in liver fibrosis [23].

MAESTRO-NAFLD-1 clinical trial (NCT04197479): This was a 52-week randomized, double-blind, placebo-controlled trial to evaluate the safety and tolerability of resmetirom 80 mg and 100 mg vs placebo. a total of 1413 patients were randomized in this trial to four arms: 80 mg resmetirom (n=327); 100 mg resmetirom (n=325); open-label 100 mg resmetirom (n=171); and placebo (n=320) [17]. The primary endpoint in this trial was the incidence of treatment-emergent adverse events (TEAEs). Of patients treated with resmetirom, 86.1-88.4% reported a TEAE, along with 81.8% of patients treated with the placebo. No specific serious TEAEs were increased in the active drug groups as compared to the placebo [17]. Similar to the MAESTRO-NASH trial, GI side effects i.e. nausea (11.9-18.2% vs 7.9%, respectively) and diarrhea (23.5%-31.2% vs 13.8%, respectively) were more frequently seen in the resmetirom arms as compared to the placebo respectively. The median duration of diarrhea in the resmetirom arms was 15-20 days independent of the dose. Resmetirom was well tolerated at both 80 and 100 mg once-daily doses over 52 weeks of treatment in this trial [17].

MAESTRO-NAFLD-OLE clinical trial (NCT04951219): The MAESTRO-NAFLD-OLE is a 52-week, open-label, Phase 3, extension, randomized controlled trial. This is an extension of the MAESTRO-NAFLD-1 trial. Patients who complete the MAESTRO-NAFLD-1 or screen fail the MAESTRO-NASH or MAESTRO-NASH-OUTCOMES trial will be selected in this study. It has two arms of resmetirom 80 mg and 100 mg once daily for 12 weeks after which all patients will receive 100 mg resmetirom for the rest of the trial. This study is currently recruiting patients and will be completed in 2026 [18]. The MAESTRO-NAFLD-1 and MAESTRO-NAFLD-OLE trials are focused on safety endpoints (treatment-emergent adverse effects and serious adverse effects). These two are non-biopsy studies wherein NASH is diagnosed and the efficacy of resmetirom is evaluated via serum and imaging biomarkers [17].

MAESTRO-NASH-OUTCOMES clinical trial (NCT05500222): The MAESTRO-NASH-OUTCOMES is a multi-national, double-blind, placebo-controlled trial studying the efficacy of 80 mg once-daily resmetirom in patients with MASH cirrhosis by measuring the time to experiencing a composite clinical outcome event. In this trial, composite clinical outcome events were defined as follows: all-cause mortality, liver transplant, and significant hepatic events, including potential hepatic decompensation events (ascites, hepatic encephalopathy, or gastroesophageal variceal hemorrhage), and confirmed increase of model for end-stage liver disease (MELD) score from <12 to ≥15. This study is currently recruiting patients and will be completed in 2027 [19]. 

## Conclusions

The clinical trials for resmetirom across phases 2 and 3 show its potential as an effective treatment for MASH, offering significant reductions in hepatic fat and improvements in liver histology while maintaining a manageable safety profile. The ongoing MAESTRO program will provide further insights into its long-term efficacy and safety in broader patient populations. Phase 2 trials have shown that resmetirom can significantly reduce hepatic fat and improve liver inflammation and fibrosis with a favorable safety profile. The ongoing Phase 3 clinical trials further aim to establish resmetirom’s long-term efficacy and safety, particularly in preventing disease progression to cirrhosis and improving liver fibrosis in patients with varying degrees of MASH severity. These trials are pivotal in confirming resmetirom’s role in the therapeutic landscape of MASH, potentially offering a novel treatment option that addresses both the metabolic and inflammatory components of the disease.
